# Phylogenetic Distribution of *csp1* Types in Aspergillus fumigatus and Their Correlates to Azole Antifungal Drug Resistance

**DOI:** 10.1128/Spectrum.01214-21

**Published:** 2021-11-17

**Authors:** Oliver Bader

**Affiliations:** a Institute for Medical Microbiology and Virology, University Medical Center Göttingen, Göttingen, Germany; Universidade de Sao Paulo

**Keywords:** *Aspergillus fumigatus*, phylogeny, azole antifungal drug susceptibility, *csp1* typing, azole antifungal drug resistance

## Abstract

In Aspergillus fumigatus, the repetitive region of the *csp1* gene is one of the most frequently used loci for intraspecies typing of this human pathogenic mold. Using PCR amplification and Sanger sequencing of only a single marker, *csp1* typing is readily available to most laboratories and highly reproducible. Here, I evaluate the usefulness of the *csp1* marker for resistance detection and epidemiologic stratification among A. fumigatus isolates. After resolving nomenclature conflicts from published studies and adding novel *csp1* types, the number of known types now adds up to 38. Their distribution mostly correlates with A. fumigatus population structure, and they are also meaningful for narrowly defined cases of azole resistance phenotypes. Isolates carrying the pandemic resistance allele TR_34_/L98H show signs of interclade crossing of strains with t02 or t04A, into the t11 clade. Furthermore, absolute differences in voriconazole MIC values between t02/t04B versus t11 TR_34_/L98H isolates indicate that the genetic background of resistance mutations may have a pivotal role in cross-resistance phenotypes and, thus, clinical outcome and environmental selection. Despite the general genetic similarity of isolates with identical *csp1* types, outcrossing into other clades is also observed. The *csp1* type alone, therefore, does not sufficiently discriminate genetic clades to be used as the sole marker in epidemiologic studies.

**IMPORTANCE** Aspergillus fumigatus is a ubiquitously distributed saprophytic mold and a leading cause of invasive aspergillosis in human hosts. Pandemic azole-resistant strains have emerged on a global scale, which are thought to be propagated through use of azole-based fungicides in agriculture. To perform epidemiologic studies, genetic typing of large cohorts is key. Here, I evaluate the usefulness of the frequently used *csp1* marker for resistance detection and epidemiologic stratification among A. fumigatus isolates. The phylogenetic distribution of *csp1* types mostly correlates with A. fumigatus population structure and is also meaningful for narrowly defined cases of azole resistance phenotypes. Nevertheless, outcrossing of *csp1* into other clades is also observed. The *csp1* type alone, therefore, does not sufficiently discriminate genetic clades and should not be used as the sole marker in epidemiologic studies.

## INTRODUCTION

Aspergillus fumigatus is a ubiquitously distributed saprophytic mold and a leading cause of invasive aspergillosis in human hosts. Invasive aspergillosis is mainly associated with immunocompromising conditions such as neutropenia, solid organ transplantation, hematopoietic stem cell transplantation, chemotherapy, or as a secondary infection to tuberculosis, influenza (1), or lately COVID-19 (2, 3). Invasive aspergillosis is *per se* a difficult-to-treat condition in already weakened patients. Most worryingly, pandemic azole-resistant strains have emerged, which can further complicate treatment (4). The origin of these resistance alleles is still under investigation, but most published studies agree that the initial mutations have likely occurred through use of azole-based fungicides in agriculture as first proposed by Snelders and coworkers (5, 6). Current data suggest that the emergent lineages may be further selected through continued fungicide use in agriculture and are amplified and propagated through composting activities where the material still contains azole fungicide residues (7). Together, this has led to the formulation of the “clonal expansion” hypothesis for some now globally distributed lineages (8).

In order to demonstrate clonality between isolates of different geographical origins and eventually trace the historic origin, genetic typing of large cohorts is key. In the absence of a working multilocus sequence type (MLST) scheme for A. fumigatus (9, 10), two other methods have gained importance as follows: multi-locus length polymorphism analysis (MLP) (also called “STRAf” for “short tandem repeat of A. fumigatus”) and sequencing of the repeat region of the *csp1* gene (“*csp1* typing”). Both methods each have specific advantages.

Its high resolution has led to the use of STRAf typing for the large-scale delineation of the A. fumigatus population on >2,000 (11) and >4,000 isolates (12). While microsatellite typing may intrinsically suffer from over interpretation of fast-changing loci, most STRAf markers were found to be sufficiently stable for population analyses (13). Together, these studies showed that the wild population of A. fumigatus largely falls into two genetic clusters, termed “A” and “B,” from which the pandemic TR_34_- and TR_46_-carrying azole-resistant linages are only small genetic subsets. They also confirmed the previously suspected clonal expansion of specific-drug-resistant lineages (8) and increasing spread of the drug resistance genes involved from clade A to clade B (12), likely through interclade crossing (14).

Less appreciated in numbers than STRAf, *csp1* typing has only been used on a smaller A. fumigatus strain collective. However, high interlaboratory reproducibility (15), broad availability of Sanger sequencing, and easy handling have kept it in use in epidemiologic studies until today. Among fungi, the principle has also been extended to Candida glabrata (16) with a two-locus scheme and has been extended by additional loci (“TRESP” [17] and “TRESPERG” [18]) in A. fumigatus. While this markedly increases discriminatory power, it has not yet been widely adopted in numbers, and these extensions will not be further explored in detail here.

Of late, numerous genome sequences of A. fumigatus have been published, which can now be exploited to further delineate the *csp1* marker and, to some degree, also the occurrence of resistance phenotypes. Here, I will focus on analyzing the phylogenetic distribution of the different *csp1* types and to what degree *csp1* typing can still contribute to handling epidemiologic questions.

## RESULTS AND DISCUSSION

### Resolving nomenclature conflicts in the literature and addition of novel *csp1* types.

The repetitive region in *csp1*, probably encoding a spacer in this extracellular cell surface protein, is composed of successions of 12-bp-long units, falling into only three to four distinct groups (Table 1). Repeat designations are indicated by an “r,” followed by numbers.

**TABLE 1:** Repeat unit definitions

No.	Classical repeat definitions		No.	Alternate and new repeat definitions	
	Nucleotide sequence^*f*^	Encoded motif		Nucleotide sequence^*f*^	Encoded motif
r09	ACT TCT GT**T** CCG	TSVP	r08	CCG ACT T**T**T **C**TC	PTFL
**r01**	**ACT TCT GTC CCG**	**TSVP**	r05	CCG ACT T**T**T GTC	PTFV
r02	ACT TCT GTC CC**A**	TSVP	r06	CCG ACT TC**A** GTC	PTSV
r10^*a*^	ACT TC**A A**TC CC**A**	TSIP	**r01**	**CCG ACT TCT GTC**	**PTSV**
r04	ACT TC**A A**TC CCG	TSIP	r21	CC**A** ACT TCT GTC	PTSV
r06	ACT TC**A** GTC CCG	TSVP	Nf2^*a*^	CCG ACT TC**C** GTC	PTSV
Nf2^*a*^	ACT TC**C** GTC CCG	TSVP	r31^*c*^	CCG AC**C** TCT GTC	PTSV
**r31**	AC**C** TCT GTC CCG	TSVP	r32^*c*^	CCG ACT **CT**T GTC	PTLV
					
**r03**	**ACT CAA AAC GCG**	**TQNA**	**r03**	**CCG ACT CAA AAC**	**PTQN**
Nf1^*b*^	ACT CA**G** AAC GCG	TQNA	r02	CC**A** ACT CAA AAC	PTQN
			Nf1^*b*^	CCG ACT CA**G** AAC	PTQN
**r07**	**ACT ACT ATT GTG**	**TTIV**	r41^*d*^	CCG ACT CAC AAC	PTHN
					
**r05**	**ACT TTT GTC CCG**	**TFVP**	r12^*e*^	CCG ACT CA**T** AAC	PTHN
r08	ACT TTT **C**TC CCG	TFLP			
			**r07**	**GCG ACT ACT ATT**	**ATTI**
			r04	GCG ACT **T**C**A** AT**C**	ATSI
			r11	GCG ACT **T**CT **G**T**C**	ATSV
			r09	GCG ACT **T**CT **G**TT	ATSV
					
			**r22**	**GTG CCA CCT CCA**	**VPPP**
			r23	GTG CC**G** CCT CC**T**	VPPP
			r24^*d*^	GTG CC**G** CCT CCA	VPPP

a Additionally described in reference 19.

b Additionally described in reference 22.

c Additionally described in this study (Aspergillus oerlinghausenensis, SRA accession SRR12143383).

d Additionally described in this study (A. fumigatiaffinis, BioProject PRJNA592352).

e Additionally described in this study (BioProject PRJNA388547).

f Bold: defining sequence for this group; red: nucleotide variations from defining sequence.

The sequential arrangement of repeats defines a set of 30 different *csp1* types to date published in the literature (see Table S1 in the supplemental material). The different *csp1* types are indicated by a “t” followed by numbers, and in a few cases extended with “A” or “B” where types appeared to be similar in early analyses. The system reflects the historic order of discovery, rather than similarity, for both individual repeats as well as *csp1* types.

Some problems in nomenclature have arisen over time. The designation t25 has been used for different *csp1* types between three studies (17, 19, 20), which are here stratified as t025C, t25G, and t25D according to the first authors’ last names. Also, t26 in reference 20 corresponds to t25G in the earlier reference (17), in which the designation t26 is used for a different succession pattern. Here, the designations t25G and t26 as defined in reference 17 are kept.

In addition, two more (t28 and t29) can be added from our own unpublished data (M. F. Mushi and O. Bader, et al.; isolates from Tanzania) and six more are evident from genome sequencing data analyzed here (t30 from SRR7418946, t31 from SRR7418923, t32 from SRR7418926, t33 from SRR10714244, t34 from SRR2954803, and t35 from SRR9067511).

Thus, there are 38 different repeat succession patterns so far, described either in the literature or here.

### A suggestion for modifying interpretation within the *csp1* typing scheme.

In the original studies developing the *csp1* typing scheme (10, 15, 21, 22), boundaries of the 12-bp repeats were chosen in a manner that one codon (=3 bp) located upstream and three codons (=9 bp) downstream of the repeat succession are further taken into account, without initially being included in the repeat succession scheme. Mainly, this was done because the −1 codon is only changed in one single *csp1* type (t04B), which was not known at the time. Definitions of repeats and numbering have already been changed twice between those early studies as more observations were made in the A. fumigatus population. Cumulating the additional data from the past 10 years shows that these 12 additional nucleotides should be included in the typing scheme by moving repeat boundary definitions one codon upstream (Table 1). While this alternate definition does not change the nature of the scheme, it does require some cosmetic changes. For one, the individual repeat sequences change (Table 1) but can be defined in a way that the numbering of the repeat succession only changes to a minute degree. The 3′ end of the repeat succession changes its nomenclature through introduction of two novel repeat types (here designated “r22” and “r23”), which now includes the 9 overhang bases. Using the alternate definitions, r01 is exclusively found at the 5′ end of the repeat succession, the one instance where r01 is found internally preceding r06 in classical patterns gives rise to a new repeat type, here designated r11. Using the alternate repeat definitions, the relationship between individual repeat succession types becomes clearer, and especially the downstream boundary of the 5′ r01 repeats becomes more clearly defined. Together, this better visualizes the different structure groups of these repeat successions (compare [classical repeat definitions] versus Table 1 [alternate repeat definitions]), although it has no implications on typing itself.

Most importantly, the designations of the individual *csp1* types are preserved, and thus published epidemiologic data do *not* require renaming.

### Ordering repeat successions.

In order to group the repeat successions by similarity with this alternate interpretation scheme, it must be noted that all *csp1* types patterns except t04B (see further below) start with 1 up to 7 r01 segments. After doubling the number of different observed *csp1* types since establishment of the original scheme, it becomes even more apparent that r01 segments do not contribute to the definition of groups, rather they appear to be deleted or inserted at moderate frequency (10), which is further supported by the phylogenetic data presented below.

**FIG 1:**
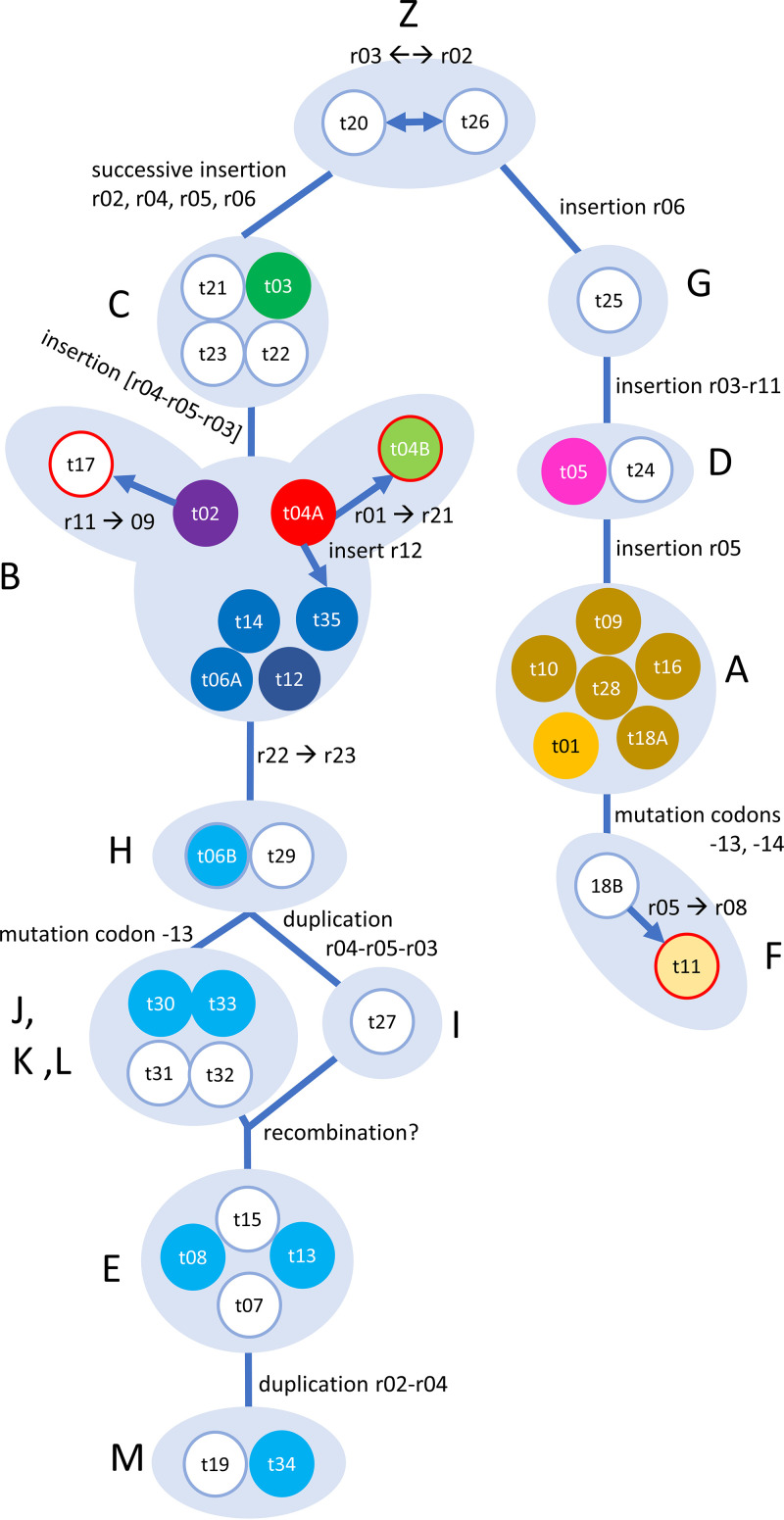
Revised model for emergence of *csp1* types. Models are based on 38 known *csp1* sequences as given in Table 2. Light blue background indicates groups as defined in Table 2. Red frames indicate three *csp1* types arisen by single SNPs not seen in any other types. Colored balls indicate *csp1* types for which genome sequences were available for analysis. *csp1* types are colored identically for better overview across both Fig. 1 and 2.

Since the establishment of the scheme, several intermediate *csp1* types have been added to the panel. The shortest *csp1* pattern “NF1” was initially found in the closely related mold *Aspergillus* (*Neosartorya*) *fischeri* (22), and derivatives of this pattern were observed only lately in A. fumigatus (t20 and t26, here designated group 0). t20 and t26 are only a synonymous A→G mutation apart interchanging r02 and r03. Starting from these, a two-winged model can be derived (Fig. 1) by successively introducing the changes either by inserting new repeats or introducing the single nucleotide polymorphisms (SNPs) observed at the 5-prime (codons −14 and −13) or 3-prime (r22 versus r23) end.

Four single *csp1* types would appear to have evolved only relatively late, three of these each by a SNP (Table 2, highlighted in light blue font), which is not observed anywhere else. These is the synonymous G→A mutation changing an r01 to r21 (t04A→t04B, in group 2C), the nonsynonymous G→C mutation changing an r05 to r08 (t18B→t11, in group 1D), and the synonymous C→T mutation changing an r11 to r09 (t02→t17, also in group 1D). The fourth, t35, presents an exception, as a novel, perhaps t02-derived, repeat (r12) is inserted after the 5′ r01 stretch and before the group-specific t02.

**TABLE 2:**
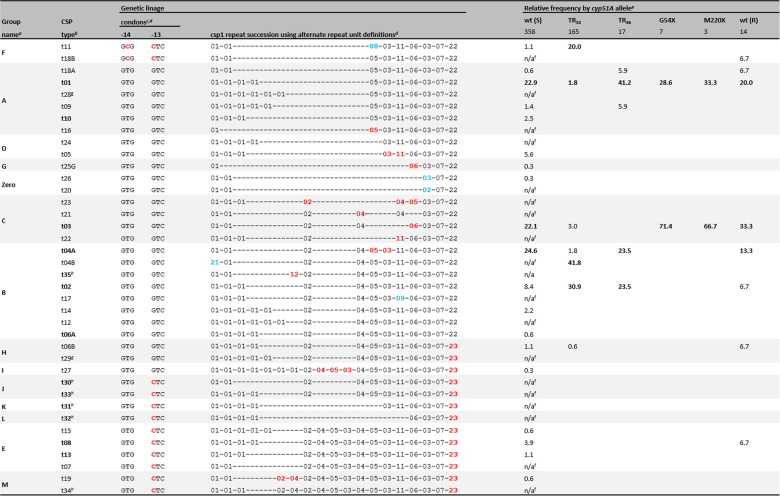
Repeat succession according to alternate repeat unit definitions versus frequency of *cyp51A* alleles

a Lineage numbering extended from Klaassen et al. (10).

b Bold indicates genome sequenced isolate(s) available (see Fig. 1 and 2).

c note that in classical *csp1* these are designated as codons -15 and -14, respectively.

d See Table 1 for classical definitions to compare to.

e S (wt), susceptible isolates with wt allele, substitutions not causing resistance, or synonymous SNPs; TR_34_, TR_34_/L98H; TR_46_, TR_46_/Y121F/(M172I)/T289A; G54, all alleles modified at codon G54; M220, all alleles modified at codon M220; R (wt), resistant isolates with wt *cyp51A* allele. Not included, TR_53_ isolates.

f N/a, not applicable. These are infrequent alleles only observed in the fraction of isolates for which no susceptibility data is available (see Table S2, group A, in the supplemental material).

g t28 and t29, unpublished *csp1* types from Tanzanian isolates (Mushi and Bader, unpublished data).

h *csp1* types newly described here, derived from genome sequencing data (see text for details). Red font indicates repeat numbers or codons different from the rest of the column and discussed in the text. Bold indicates percentages are >10% among susceptible isolates (arbitrary cutoff for highlighting only). Light blue font indicates that repeats are only a single SNP apart.

### *csp1* types present in the azole-susceptible A. fumigatus wild population.

To date, *csp1* typing has been used by 17 different studies either individually or as part of TRESP/TRESPERG typing (10, 14, 17–20, 22–32), looking at both drug-susceptible and -resistant isolates. Unfortunately, the four early studies (10, 22, 23, 33) and the most recent one (20) did not (yet) test their isolates’ drug susceptibility. While these data sets create significant insight into the *csp1* alleles present in the A. fumigatus population, they cannot unequivocally be used to analyze progression of drug resistance therein.

In order to at least partially compensate for this, an additional set of 122 azole-susceptible isolates from Germany taken from our own clinical study (25) was *csp1* typed here (contained within Table S2 in the supplemental material). These were chosen to reflect the maximum geographical diversity present in this particular collection.

Taken together, there are 358 susceptible *csp1*-type isolates as a basis for analyses. Indeed, both cumulated data sets (Table 2, groups A and B) are highly similar in composition, with the peculiar exception of t04B, which is discussed further below. The majority (82.6%) of published isolates stems only from five distinct *csp1* types (t01 [22.9%], t02 [8.4%], t03 [22.1%], t04A [24.6%], and t05 [5.6%]), which are not very surprisingly also the ones first described, and, with the exception of t05, the ones forming the vast majority of publicly available genome sequencing data.

### Overall correlation of *csp1* types with SNP-based phylogeny.

Phylogenetic distribution of the *csp1* types across 210 publicly available sequenced genomes of A. fumigatus (Fig. 2A) suggests good, although not full, correlation with the overall population structure. In the main body of the resulting SNPome-based tree (Fig. 2B), each major subtype (t01, t02, t03, t04A, and possibly t05 and t11) is represented by a branch that is either specific or at least highly enriched for isolates with that particular subtype and its r01 number derivatives. Also, the *csp1* SNPs observed at the 5-prime end of the repeat region in codon −13 and at the 3-prime end in r22 versus r23 correlate with phylogeny as described further below.

**FIG 2:**
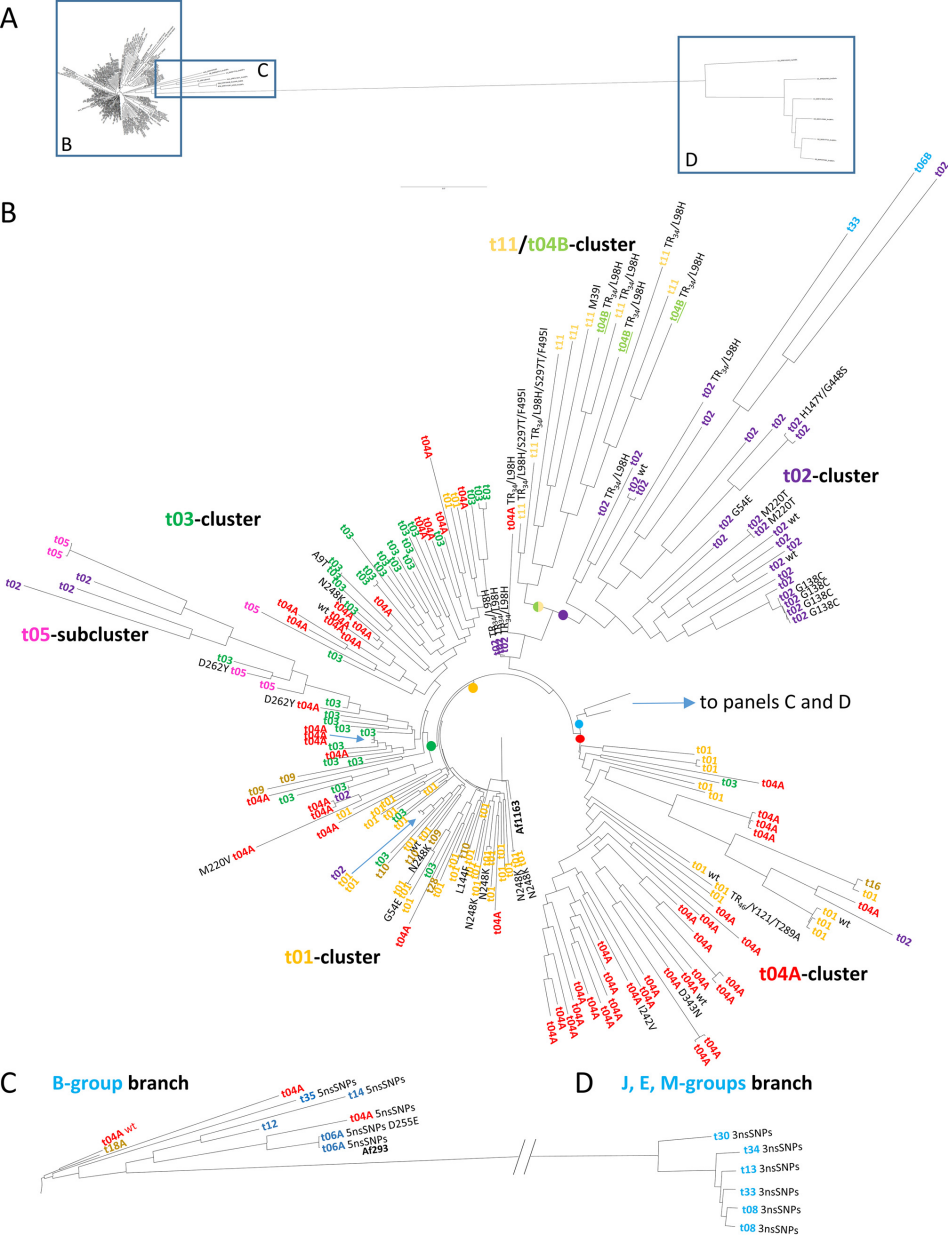
Phylogenetic placement of *csp1* types and *cyp51A* alleles. (A) Midpoint rooted phylogenetic tree constructed from whole SNPomes of 210 shotgun genome sequences. Main body of phylogenetic tree (B), 5 nsSPNs branch (C), and 3 nsSNPs branch (D). *csp1* types are colored identically for better overview across both Fig. 1 and 2. *cyp51A* isoforms of resistant isolates are given after *csp1* types, where “wt” denotes azole-resistant isolates with unaltered (compared to Af1163) *cyp51A* sequence. The SNP alignment and full tree in Newick format are available in File S1 and S2 in the supplemental material, respectively. SRA accession numbers are given in Fig. S1 in the supplemental material.

Isolates from the groups J, E, and M (Fig. 1) are segregated together into a separate branch (Fig. 2D). These also form a subgroup of *cyp51A* alleles that encode a set of three distinct amino acid substitutions (F46Y/M172V/E427K) with no apparent effect of azole susceptibility (termed lineage 4 in reference 34). The foot of this branch (Fig. 2C), which includes the widely used reference strain AF293, harbors *csp1* types from group B (t04A, t06A, t12, t14, and t35). This is in line with the model predicting linear elongation of *csp1* types over the course of evolution, starting from group B leading over groups H to L, and eventually to E and M (Fig. 1). All of these bear the terminal r23 instead of the r22 repeat (Table 2), and most of these isolates also have a *cyp51A* allele, which encodes for five amino acid substitutions (F46Y/M172V/N248T/D255E/E427K), again with no apparent effect of azole susceptibility. This lineage is termed lineage 3 in reference 34, and those conclusions are supported by the analyses with independently sequenced isolates shown here.

*csp1* types that only differ in the number of r01 repeats also mostly cluster closely together, e.g., t08 and t13 in group E; t06A, t35, t12, t14, and partially t04A from group B (notably except t02 and t04B); and t10, t09, t28, t16, and t01 from group A. This further supports earlier hypotheses that the number of leading r01 repeats has no value for asserting phylogenetic subgroups, as these numerical aberrations arise or vanish more quickly (10).

The reference strain Af1163 here used for constructing the phylogeny is of *csp1* type t01. Consequently, the cluster almost exclusive for *csp1* types of the A group (t01, t09, t10, t28) is found at the midpoint root of the phylogenetic tree (Fig. 2B). At the other most distantly placed arms within the main tree body, well-segregated clusters of t02 and t04 are found. In contrast, clusters for *csp1* types t03, t05, and t11 are less well defined, although they are apparent on the tree. For both, the t05 and t11 clusters, this may be due to the still low number of isolates that could be included in the analysis. In contrast, for t03, it is highly evident that this marker has also migrated near and into the other clusters, e.g., to the bottom of the t05 branch. Similarly, while there is a distinct cluster for t04, isolates with this *csp1* type are also found in all other parts of the tree.

A. fumigatus has been shown to possess a sexual cycle (35) and a panmictic population structure (36). Sexual recombination explains why there is no strict segregation of particular *csp1* types into distinct branches in the phylogenetic tree (Fig. 2A), including the appearance of isolated strains with deviating *csp1* types near clusters. This is further supported by the observation that genetic distances tend to grow when *csp1* types appear near or within clusters of other types. An example would be the appearance of t02 within the t05 branch or t04A cluster and isolates from the otherwise distant groups H (t06B) and J (t33) in a separate and elongated branch within the t02 cluster.

### Correlation of *csp1* types with azole drug-resistant lineages.

The most prevalent and best-understood resistance mechanisms toward azole-based antifungals or fungicides are mutations in the locus for the target enzyme CYP51A. In A. fumigatus, *cyp51A* is one of two paralogous genes encoding key enzymes in the biosynthesis of the membrane sterol ergosterol. For the *cyp51B* locus, only a single resistance mutation is known (37). By far, the more frequent resistance mutations in *cyp51A* either alter drug interactions of the protein or increase transcriptional levels. Within the cumulated subset of isolates for which both a *csp1* type and the *cyp51A* allele are known, *cyp51A* resistance mutations are present in 93% of resistant isolates (Table 2). This is, however, different in other data sets where non-*cyp51A* mechanisms can make up to half of resistant isolates. Within the small group of resistant but *cyp51A* wild-type (wt) isolates with a known *csp1* type (*n* = 14) (Table 2), the relative distribution follows roughly the one for susceptible isolates, offering no particular correlation.

In the reference genomes of Afu293 and A1163, *csp1* localizes to chromosome 3, whereas *cyp51A* is encoded on chromosome 4. This already suggests a lack of genetic linkage between the two loci, and indeed azole-resistant TR_34_/L98H isolates of *csp1* types t02, t04B, and t11 experimentally crossed with susceptible cyp51A wt strains of *csp1* types t05 or t03 rendered resistant TR_34_/L98H progeny of *csp1* types t03 and t05 (14). Recombination has also been observed in a subset of Dutch and Indian ARAf isolates (36). This demonstrates that particular *cyp51A*-based mechanisms do not need to be restricted to specific *csp1* types or be exclusively correlated to any other molecular markers used for intraspecies typing.

Epidemiologic data cumulated from the literature and obtained in this study (Table 2), however, do allow for some reflections on *csp1* types and resistance profiles with respect to their phylogenetic distribution.

On a global level, there is no strict categorical positive correlation of specific *csp1* types with a particular resistance mutation or even resistance at all. Therefore, determining the *csp1* type has unfortunately no value for detection of resistance per se. However, vice versa, some mutations are indeed associated only with a reduced set of *csp1* types. This is particularly true for the two *cyp51A* alleles F46Y/M172V/E427K and F46Y/M172V/N248T/D255E/E427K, which are not implicated as drivers of drug resistance but appear as wild-type alleles in specific clusters at the distant branches of the population.

Among known resistance-conferring substitutions, N248K is most frequently found in Japan (38), which may indicate its recent emergence in Asia. Genome sequencing data of three Portuguese isolates suggest the N248K substitution to be present in a small subset of t01 strains only (Fig. 2, t01 cluster), further supported by some other isolates in this cluster from other studies. However, it also appears at least once in the t03 cluster from another sequencing study (39), indicating either spread to, or coemergence in, other lineages.

Cyp51A alleles with mutations at G54 or M220 are historically viewed as “clinically induced” but have subsequently also been found in environmental isolates (26, 28, 40). So far, it is unknown whether these substitutions arise and propagate in the environment as well or if they were shed from patients. G54 and M220 substitutions have only been associated with *csp1* types t01 and t03 in the literature (Table 2) but are also found in t02 and t04A in genome sequencing data (Fig. 2), indicating their unrelated emergence. Isolates with the G138C substitution appear as a subbranch in the t02 cluster, which may represent a sampling bias, as all of these isolates stem from the same study. For the rarer resistance mutations, either no, or only very limited, data exist (see Table S2G in the supplemental material), which renders any analyses mere speculation at this point.

This is, however, different for the most frequently observed resistance allele TR_34_/L98H. TR_34_/L98H, TR_46_/Y121F/T289A, and several others less frequently observed alleles, such as TR_53_/…, TR_126_/…, etc., differ by additional alterations in the promoter, differentially modulating *cyp51A* transcription in response to drug exposure (41). STRAf typing already suggests a different origin of these strains (12). This is in agreement with *csp1* typing, which finds mainly t04B (∼40%), t02 (∼30%), and t11 (∼20%) co-occurring with the TR_34_/L98H, and t01 (∼40%), t02 (∼25%), and t04A (∼25%) with the TR_46_/Y121F/T289A allele (Table 2). The only available genome sequenced strain with a TR_46_ repeat is placed with other *csp1* type t01 isolates, however, at the base of the t04 cluster.

Most interestingly, resistant isolates with the TR_34_/L98H *cyp51A* allele form a branch by themselves, connected at the basis with the t02 cluster. This is independent of their *csp1* type and intermingles with the azole susceptible t11 isolates included in the study (Fig. 2, t11 cluster). At the bottom of the branch, three nearly clonal TR_34_/L98H isolates of the t02 type are found, and this is also the place where five further isolates reside, which were removed from the phylogenetic analysis as they produced only identical SNPomes to another strain of this cluster. The entire cluster of Indian isolates stems from a single study (36), and while it may represent a sampling bias, this would also be in high accordance with the “clonal expansion” hypothesis (8) for the TR_34_/L98H allele.

Within the larger branch of what would be the t11 cluster, t04B forms a peculiar case, as this *csp1* type has not yet been associated with any other *cyp51A* alleles than TR_34_/L98H and represents the largest single group of azole drug-resistant TR_34_/L98H isolates (Table 2). Another subgroup is formed by the TR_34_/L98H/S297T/F495I allele, which is predominantly found in Asia (32, 42) but for which isolated reports from South America and Europe (43) also exist. These isolates appear highly related by STRAf typing (43), and those originating from China display only t01 and t11 *csp1* types so far (32).

Development of drug resistance traits often coincides with fitness defects, which select for secondary compensatory mutations inside the host (44) or potentially the environment. However, no fitness defects of *cyp51A* mutations at G54 or M220, or for the TR_34_/L98H allele, could be demonstrated so far (45–47).

The higher individual genetic distance of TR_34_/L98H isolates, with the exception of those in the clonal group at the bottom of the branch, indicates that the genetic background of t02 combined with t11 isolates may stabilize the resistance mutation in TR_34_/L98H isolates and allow independent genetic material on chromosome 3 (harboring the locus of the *csp1* marker) and others to be introduced at higher rates into these lineages.

Camps et al. (14) observed that already the earliest Dutch TR_34_/L98H isolates (collected in 1998) were t11, while t04B and t02 were only found in strains isolated 2 and 4 years later, respectively. In those data, the absolute frequency of t02 and t04B increased over the years and also peaked in relative terms over t11 at the end of the observation period (in 2007). This ratio of *csp1* types was also found in TR_34_/L98H isolates from Germany (clinical isolates sampled in 2010/11 and environmental isolates in 2013/14 [26]).

**FIG 3:**
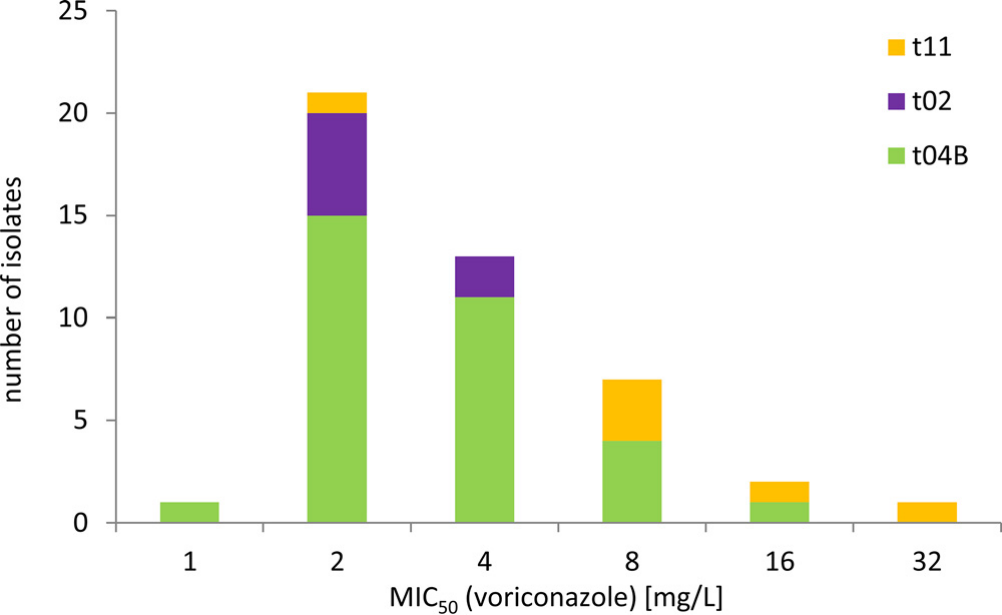
Voriconazole MIC values of German TR_34_/L98H isolates stratified by *csp1* type.

Retrospective stratification of MIC data for TR_34_/L98H isolates from two German studies (25, 26) by *csp1* type shows that those t11 isolates actually had a 4- to 8-fold higher average voriconazole MIC than those of t02 (U = 4.5, *Z* = −2.28571 at threshold of *P* being <0.5, Mann-Whitney U test) while those of t04B isolates were more broadly distributed (Fig. 3). This indicates that the genetic background of particular resistance mutations may indeed have an influence on the MIC values of cross-resistance phenotypes and thus clinical outcome as well as selection in the environment. In addition, it may also explain why for some mutations cumulated MIC data show a very broad distribution and partial incoherence between different studies (48).

Next to clonal spread of the resistant lineages under selection pressure, gradual outcrossing of resistance traits into the broader population is therefore to be expected. In the future, it will be interesting to investigate why especially those TR_34_/L98H isolates of the *csp1* type t04B appear to make up the majority of the pandemic isolates.

### Conclusions.

Due to its low threshold in terms of laboratory work, marker gene sequencing is attractive for strain typing. The *csp1* marker has clear correlations to the population structure of A. fumigatus, likely to the only infrequent sexual reproduction of this species. This is especially true for the rarer *csp1* types. Still, the correlation is not absolute, and, unfortunately, it only bears low significance for the *ab initio* detection of resistance. Using the current database, no strong conclusions on resistance types other than TR_34_/L98H can be made. In the phylogenetic subgroup of TR_34_/L98H isolates, markers t11 and t04B are highly overrepresented but others (t02, t04A) are not, and there the *cyp51A* locus resistance allele is more predictive for phylogeny than the *csp1* type. The origin of the t04B *csp1* type remains unclear, as it has not yet been found in non-TR_34_/L98H isolates and is already distributed within the t11-TR_34_/L98H cluster. Future epidemiologic analyses should not rely on *csp1* typing as a sole genetic typing marker.

## MATERIALS AND METHODS

### Sanger sequencing of the *csp1* gene from German clinical A. fumigatus isolates.

A total of 122 isolates were taken from cryostocks prepared during a previous study (25). Cells were cultured in 5 ml liquid Sabouraud’s medium (bioMérieux) at room temperature on a turning wheel. DNA was prepared using beating of the cells with glass beads in a FastPrep FP120 machine in phenol-chloroform at speed setting 4 for 30 s. The *csp1* gene was PCR amplified as described before (21) and Sanger sequenced using the reverse primer (Seqlab Microsynth). *csp1* repeat patterns were matched to the sequence derived from the trace file using Geneious Prime V 2020.0.3.

### *csp1* and *cyp51A* genotyping from whole-genome sequencing data.

As an initial database, publicly available whole-genome sequence data for A. fumigatus labeled as “paired end” and “random library prep” were downloaded from NCBIs short read archive in February 2021 (see Table S3 in the supplemental material). This identified 15 different BioProjects listed in SRA from which data were included in this study (Table 3), amounting to 220 sequencing runs fulfilling the criteria as outlined blow.

**TABLE 3:** Genome sequencing data included in phylogenetic analysis^*a*^

BioProject accession no.	Study rationale	No. runs	No. included	Reference
PRJNA67101	ARAf strains	26	24	51
PRJEB8623	Phylogeny of azole-resistant A. fumigatus	24	19	36
PRJNA427336	Thoracic transplant recipients	2	2	52
PRJNA477519	A. fumigatus phylogeny	28	15	53
PRJNA388547	Outdoor tropical air in Singapore	1	1	54
PRJNA592352	A. fumigatus sibling species	15	4	55
PRJNA595552	Environmental ARAf	64	51	56
PRJNA673120	SARS-CoV infections	4	4	3
PRJNA638646	Itraconazole sensitivity	68	68	39
PRJNA659567, PRJNA319359	Environmental microbiome aboard International Space Station	3	2	57
PRJNA671765	Amphotericin B sensitivity	12	11	
PRJNA575185	Testing DNA extraction kits	36	1	
PRJNA390160	Recurrent aspergillosis	8	7	
PRJNA298653	Peruvian rain forest soil	1	1	

a Details on grounds for excluding individual data sets are given in Table S3 in the supplemental material.

For each data set, excluding those concomitantly identified for non-A. fumigatus Aspergillus species (Table 4), trimmed reads were mapped against the full genome sequence of reference strain Af1163 using BWA-MEM and fold-coverage calculated using bedtools.

**TABLE 4:**
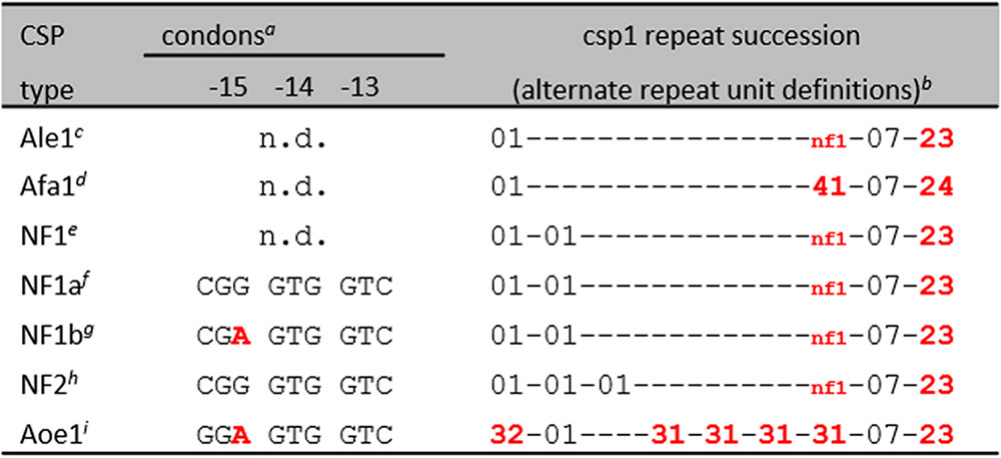
Repeat succession in non-A. fumigatus *Aspergillus* species

^*a*^Note that in classical *csp1* typing, these are codons −16, −15, and −14, respectively.

^*b*^See Table 1 for sequences.

^*c*^A. lentulus from PRJNA592352 (all isolates).

^*d*^A. fumigatiaffinis from PRJNA592352 (all isolates).

^*e*^*A*. (*Neosartorya*) *fischeri* (22).

^*f*^*A. fisheri* SRR10092049 and SRR10092050 (58).

^*g*^*A. fisheri* SRR11363404 (58).

^*h*^*A. fisheri* SRR11363405 (58).

^*i*^Aspergillus oerlinghausenensis SRR12143383 (58).

For evaluation of *csp1* types and *cyp51A*, trimmed reads were mapped against an artificial t08 type *csp1* as well as the *cyp51A* sequence and mapped reads extracted (BWA filter) using the https://usegalaxy.eu environment. All subsequent operations for *cyp51A* and *csp1* sequences were carried out in Geneious Prime using the built-in algorithms: *cyp51A* reads were mapped to an annotated reference (48). *csp1* reads were *de novo* assembled (inbuilt Geneious assembler) with no gapping allowed to force repeat assembly and more stringent mapping parameters (maximum mismatches per read = 4%; maximum ambiguity = 4) than the standard setting. To determine the *csp1* type, *csp1* repeat patterns (Table 1) were matched to the derived consensus sequences. High-quality data also allowed detection of sequencing data from probable mixed cultures by occurrence of multiple well-covered *csp1* contigs. Shotgun sequences with a read length of <200 bp turned out to be mostly insufficient for *csp1* type determination and were excluded (exclusion indicated in Table S3).

A similar approach to derive the STRAf type from genome data was initially tested but discarded as even read lengths of 250-bp paired-end runs were insufficient for determination of repeat types, as they did not cover the start and end of the longer repeats in a single sequence. This confirms earlier observations that assembly loci with highly repeated short sequences may be possible but are often very difficult from genome sequencing data (16).

### Genome SNP-based clustering analysis.

Data sets with a raw genome coverage of <30-fold were excluded from SNP analyses unless they represented very rare *csp1* types (indicated in Table S3). For all 220 A. fumigatus genomes where both a reliable *cyp51A* sequence and a *csp1* type could be determined, the whole SNPome was determined using Snippy V4.4.3 with the genome of strain A1163 as a reference (49), and the core SNPome using Snippy core, both as implemented in the https://usegalaxy.eu environment (File S1 in the supplemental material). Ten read archives were excluded because they produced SNPomes fully identical to others in the cohort. A tree was generated using RAxML 8.2.11 (bootstrapping with 210 replicates, GTR GAMMA nucleotide substitution model) from the resulting core SNP alignment encompassing 209,729 SNPs from 210 mappings. The full Newick format tree is available as File S2 in the supplemental material.
