# Supplementary material for: Phylogenetic Distribution of csp1 Types in Aspergillus fumigatus and Their Correlates to Azole Antifungal Drug Resistance
Source: Microbiol Spectr. 2021 Nov 17;9(3):e01214-21. doi: 10.1128/Spectrum.01214-21 (PMC8597649; doi:10.1128/Spectrum.01214-21)
Supplement: SUPPLEMENTAL FILE 1 — Supplemental material. Download SPECTRUM01214-21_Supp_1_seq5.pdf, PDF file, 3.4 MB [file spectrum01214-21_supp_1_seq5.pdf]

SUPPLEMENT to

Phylogenetic distribution of csp1-types in *Aspergillus fumigatus* and their correlates to azole antifungal drug resistance

Oliver Bader

Institute for Medical Microbiology, University Medical Center Göttingen, Kreuzbergring 57, 37075 Göttingen, Germany

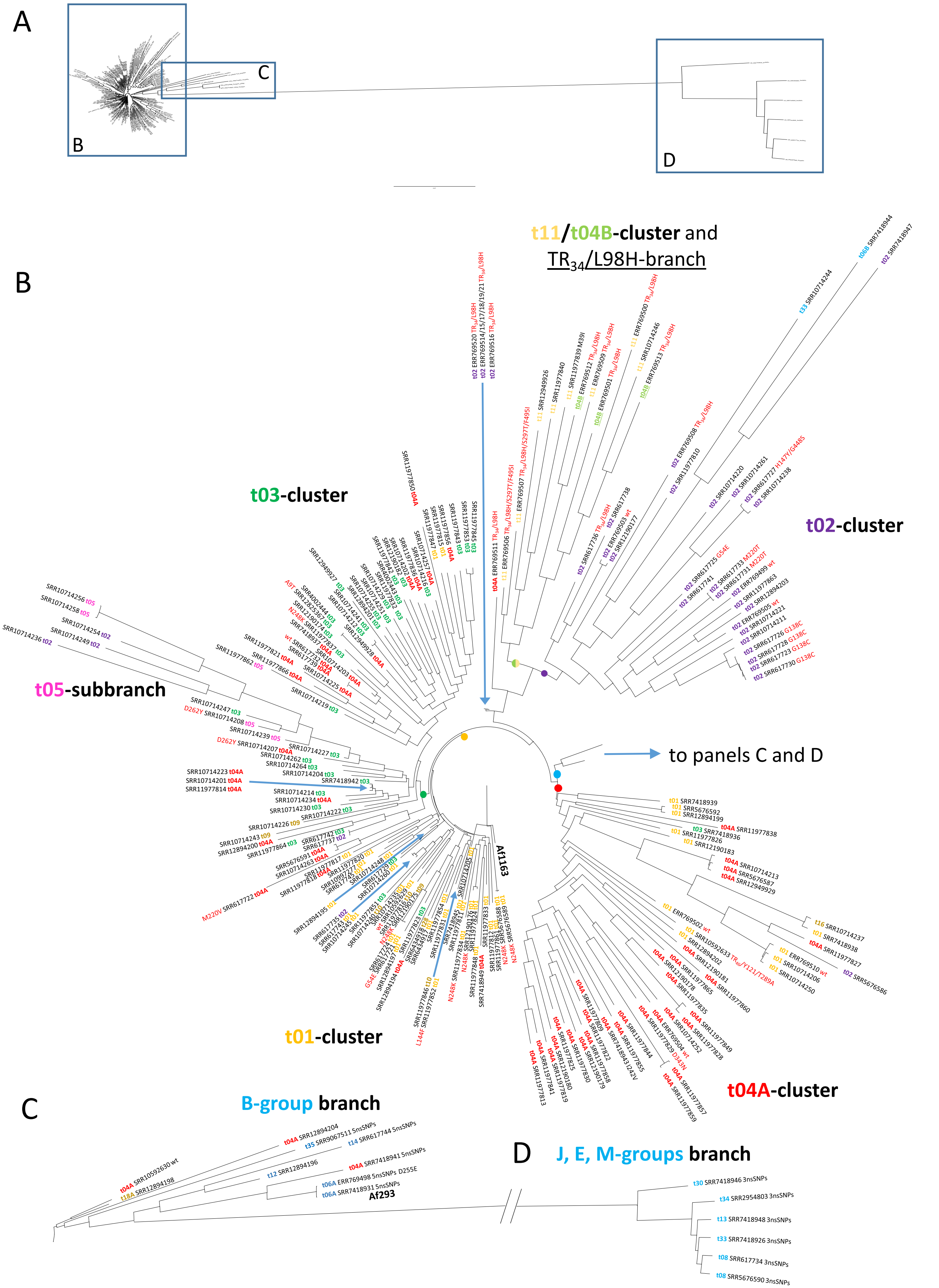

Supplementary figure 1: phylogenetic placement of csp1 types and cyp51A alleles, including SRA accession numbers. Rooted phylogenetic tree constructed from whole SNPomes of 211 shotgun genome sequencesL. bold: reference genomes. cyp51A isoforms are given after csp1 types as compared to the A1163 reference. “wt” denotes resistant isolates without changes in Cyp51A sequence. Coloured balls are placed at branch intersections used for respective cluster definition.

Supplement

Supplementary Table 1: The 30 CSP types published to date among *Aspergillus fumigatus* isolates using classical repeat unit definitions

CSP type	Condon -15 -14 -1	Tandem repeat succession using *classical repeat unit definitions*	Condon +1 +2 +3	First reported in reference
t11	G**C**G **C**TC CCG	01-01-------------------------------------**08**-03-01-06-03-07	CCA CCT CCA	(1)
t18B	G**C**G **C**TC CCG	01-01-------------------------------------05-03-01-06-03-07	CCA CCT CCA	(2)
t18A[a]	GTG GTC CCG	01-01-------------------------------------05-03-01-06-03-07	CCA CCT CCA	(1)
t09	GTG GTC CCG	01-01-01-01-01----------------------------05-03-01-06-03-07	CCA CCT CCA	(1)
t01	GTG GTC CCG	01-01-01-01-------------------------------05-03-01-06-03-07	CCA CCT CCA	(1, 3)
t10	GTG GTC CCG	01-01-01----------------------------------05-03-01-06-03-07	CCA CCT CCA	(1)
t16	GTG GTC CCG	01--05-03-01-06-03-07	CCA CCT CCA	(1)
t24	GTG GTC CCG	01-01-01-01----------------------------------03-01-06-03-07	CCA CCT CCA	(2)
t05	GTG GTC CCG	01-01-01-------------------------------------03-01-06-03-07	CCA CCT CCA	(1, 3)
t25G[b]	GTG GTC CCG	01---06-03-07	CCA CCT CCA	(4)
t26[b]	GTG GTC CCG	01-01---03-07	CCA CCT CCA	(4)
NF1	GTG GTC CCG	01-01---**nf1**-07	CCA CCT CCA	(5)
t20	GTG GTC CCG	01----------------02----------------------------------03-07	CCA CCT CCA	(5)
t22	GTG GTC CCG	01-01-------------02-------------------------03-01-06-03-07	CCA CCT CCA	(2)
t03	GTG GTC CCG	01----------------02----------------03-04-**06**----------03-07	CCA CCT CCA	(1, 3)
t21	GTG GTC CCG	01----------------02----------------03-04-**04**----------03-07	CCA CCT CCA	(2)
t23	GTG GTC CCG	01-01-------------02----------------03-04-05----------03-07	CCA CCT CCA	(2)
t04A	GTG GTC CCG	01----------------02----------------03-04-05-03-01-06-03-07	CCA CCT CCA	(1, 3)
t04B	GTG GTC CC**A**	01----------------02----------------03-04-05-03-01-06-03-07	CCA CCT CCA	(1)
t02	GTG GTC CCG	01-01-------------02----------------03-04-05-03-01-06-03-07	CCA CCT CCA	(1, 3)
t25D[b]	GTG GTC CCG	01-01-------------02----------------03-04-05-03-01-**01**-03-07	CCA CCT CCA	(6)
t25C[b]	GTG GTC CCG	01----------------02----------------03-04-05-03-01-**nf2**-03-07	CCA CCT CCA	(7)
t17	GTG GTC CCG	01-01-------------02----------------03-04-05-03-**09**-06-03-07	CCA CCT CCA	(1)
t14	GTG GTC CCG	01-01-01-01-------02----------------03-04-05-03-01-06-03-07	CCA CCT CCA	(1)
t12	GTG GTC CCG	01-01-01-01-01----02----------------03-04-05-03-01-06-03-07	CCA CCT CCA	(1)
t06A	GTG GTC CCG	01-01-01----------02----------------03-04-05-03-01-06-03-07	CCA CCT CCA	(1, 3)
t06B	GTG GTC CCG	01-01-01----------02----------------03-04-05-03-01-06-03-07	CC**G** CCT CC**T**	(5)
t27	GTG GTC CCG	01-01-01-01-01-01-02-------03-04-05-03-04-05-03-01-06-03-07	CC**G** CCT CC**T**	(4)
t15	GTG **C**TC CCG	01-01-01-01-------02-------03-04-05-03-04-05-03-01-06-03-07	CC**G** CCT CC**T**	(1)
t08	GTG **C**TC CCG	01-01-01----------02-------03-04-05-03-04-05-03-01-06-03-07	CC**G** CCT CC**T**	(1)
t13	GTG **C**TC CCG	01-01-------------02-------03-04-05-03-04-05-03-01-06-03-07	CC**G** CCT CC**T**	(1)
t07	GTG **C**TC CCG	01----------------02-------03-04-05-03-04-05-03-01-06-03-07	CC**G** CCT CC**T**	(1)
t19	GTG **C**TC CCG	01----------------02-03-**10**-03-04-05-03-04-05-03-01-06-03-07	CC**G** CCT CC**T**	(5)

[a] CSP type t18A was previously designated only as t18. [b] there is naming conflict between refs (4), (7), and (6), see text for details.

Supplementary Table 2: detailed *csp1*-type-stratified epidemiologic data

origin[a]	t01	t02	t03	t04A	t04B	t05	t06A	t06B	t07	t08	t09	t10	t11	t12	t13	t14	t15	t16	t17	t18A	t18B	t19	t20	t21	t22	t23	t24	t25C	t25D	t25G	t26	t27	total	reference
**A)** isolates without documented susceptibility testing																																		
NL	78	21	23	36	2	19	1		1	7	4	2	4	1	1	6	1	1	1														209	(1)
OZ	26	9	29	35		2	3	1			1	4			2	1			1	2		5	1										122	(5)
OZ	3	1	2	4																													10	(8)
CN	19	16	33	51		2	5	9				6		3		3			1		3			1	8	1	1						162	(2)
MX	7	7	4	21				8				3			3	1																	54	(6)
AR	2	3	8	12												1													1		2		29	(6)
PE	2			1																													3	(6)
FR			1	3																													4	(6)
**sum**	**137**	**57**	**100**	**163**	**2**	**23**	**9**	**18**	**1**	**7**	**5**	**15**	**4**	**4**	**6**	**12**	**1**	**1**	**3**	**2**	**3**	**5**	**1**	**1**	**8**	**1**	**1**				2		**593**	
%	25.4	10.6	18.6	30.2	0.4	4.3	1.7	3.3	0.2	1.3	0.9	2.8	0.7	0.7	1.16	2.2	0.2	0.2	0.6	0.4	0.6	0.9	0.2	0.2	1.5	0.2	0.2				0.4			
**B)** azole susceptible isolates																																		
NL	15	4	7	16		2				5	1	1			2	1				1													55	(9)
ES	24	11	15	32		3		4		4	2	1	3		1	4	1			1		2								1	1	1	111	(4, 10)
CN	57	11	21	38		1	3	1				7		1		1			1	4	4				2						1		153	(7)
IR	19	1	13	24			2			1		3																					63	(11)
DE	***24***	***14***	***43***	***13***		***14***				***3***	***2***	***4***			***1***	***3***	***1***																***122***	***this study***
UK			1	3		1				1																							6	(12)
**sum**	**82**	**30**	**79**	**88**		**20**	**2**	**4**		**14**	**5**	**9**	**3**		**4**	**8**	**2**			**2**		**2**						**1**			**1**	**1**	**357**	
%	22.9	8.4	22.1	24.6		5.6	0.6	1.1		3.9	1.4	2.5	0.9		1.1	2.2	0.6			0.6		0.6						0.3			0.3	0.3		

Supplementary Table 2 (continued)

origin[a]	t01	t02	t03	t04A	t04B	t05	t06A	t06B	t07	t08	t09	t10	t11	t12	t13	t14	t15	t16	t17	t18A	t18B	t19	t20	t21	t22	t23	t24	t25C	t25D	t25G	t26	t27	total	reference
**C)** azole resistant isolates with TR_{34}/L98H																																		
NL		12			20								23																				55	(9)
ES		9		1																													10	(4, 10)
DE	1	10		1	42								8																				62	(13, 14)
IR		5	2	1				1																									9	(11)
TZ	1	2																															3	(15)
TH					7								1																				8	(16)
UK	1	9	2																														12	(12)
CH		4	1										1																				6	(17)
CN		3		1			1						1																				6	(18)
CN		5																															5	(19)
CN[b]	2			1									5																				8	(19)
**sum**	**5**	**56**	**5**	**4**	**69**			**1**					**38**																				**178**	
%																																		
**D)** azole resistant isolates with TR_{46}-repeat alleles																																		
ES	1																																1	(4, 10)
DE	5	1		4																													10	(13, 14, 20)
UK	1	3									1									1													6	(12)
CN	4			2																													6	(19)
**sum**	**11**	**4**		**6**							**1**									**1**													**23**	
%	41.2	23.5		23.5							5.9									5.9														
**E)** resistant isolates with substitutions at M220																																		
DE	1		2																														3	(13, 14)
%	33.3		66.7																															

Supplementary Table 2 (continued)

origin[a]	t01	t02	t03	t04A	t04B	t05	t06A	t06B	t07	t08	t09	t10	t11	t12	t13	t14	t15	t16	t17	t18A	t18B	t19	t20	t21	t22	t23	t24	t25C	t25D	t25G	t26	t27	total	reference
	**F)** resistant isolates with substitutions at G54																																	
TH			3																														3	(16)
CH	1																																1	(17)
DE	1		2																														3	(14)
ES				1																													1	(21)
**sum**	**2**		**5**	**1**																													8	
%	41.2	23.5		23.5							5.9									5.9														
	**G)** resistant isolates with wt cyp51A																																	
IR	1		1	2																1	1												6	(11)
DE	2	1	4					1		1																							9	(14)
ES				1																													1	(21)
**sum**	**3**	**1**	**5**	**3**				**1**		**1**										**1**	**1**												**16**	
%	18.7	6.3	31.3	18.7				6.3		6.3										6.3	6.3													
	**H)** resistant isolates with other substitutions in cyp51A or undisclosed mechanisms																																	
DE	1																																1	(14)
NL	4	1	1	1																													7	(9)
ES	2		2	2																													6	(4, 10)

[a] country codes: NL: The Netherlands; OZ: Australia; CN: China, MX: Mexico, AR: Argentina; PE: Peru, ES: Spain; IR: Ireland; DE: Germany; UK: United Kingdom; TZ: Tanzania; TH: Thailand; CH: Switzerland; USA: United States of America; [b] allele type TR_{34}/L98H/S297T/F495I

Supplementary Table 3: details on genome data set

(excel sheet) available from https://github.com/oliverbader/Aspergillus_fumigatus_cyp51A

Supplementary file 1: SNP alignment

(.fasta.gz file) available from https://github.com/oliverbader/Aspergillus_fumigatus_cyp51A

Supplementary file 2: Data for Newick-format phylogenetic tree

(.nhx file) available from https://github.com/oliverbader/Aspergillus_fumigatus_cyp51A

References

1. Klaassen CH, de Valk HA, Balajee SA, Meis JF. 2009. Utility of CSP typing to sub-type clinical *Aspergillus fumigatus* isolates and proposal for a new CSP type nomenclature. J Microbiol Methods 77:292-6.
2. Gao LJ, Sun Y, Wan Z, Li RY, Yu J. 2013. CSP typing of Chinese *Aspergillus fumigatus* isolates: identification of additional CSP types. Med Mycol 51:683-7.
3. Balajee SA, Tay ST, Lasker BA, Hurst SF, Rooney AP. 2007. Characterization of a novel gene for strain typing reveals substructuring of *Aspergillus fumigatus* across North America. Eukaryot Cell 6:1392-9.
4. Garcia-Rubio R, Gil H, Monteiro MC, Pelaez T, Mellado E. 2016. A New *Aspergillus fumigatus* Typing Method Based on Hypervariable Tandem Repeats Located within Exons of Surface Protein Coding Genes (TRESP). PLoS One 11:e0163869.
5. Kidd SE, Nik Zulkepeli NA, Slavin MA, Morrissey CO. 2009. Utility of a proposed CSP typing nomenclature for Australian *Aspergillus fumigatus* isolates: Identification of additional CSP types and suggested modifications. J Microbiol Methods 78:223-6.
6. Duarte-Escalante E, Frias-De-Leon MG, Martinez-Herrera E, Acosta-Altamirano G, de Paz ER, Resendiz-Sanchez J, Refojo N, Reyes-Montes MDR. 2020. Identification of CSP Types and Genotypic Variability of Clinical and Environmental Isolates of *Aspergillus fumigatus* from Different Geographic Origins. Microorganisms 8.
7. Chen Y, Lu Z, Zhao J, Zou Z, Gong Y, Qu F, Bao Z, Qiu G, Song M, Zhang Q, Liu L, Hu M, Han X, Tian S, Zhao J, Chen F, Zhang C, Sun Y, Verweij PE, Huang L, Han L. 2016. Epidemiology and Molecular Characterizations of Azole Resistance in Clinical and Environmental *Aspergillus fumigatus* Isolates from China. Antimicrob Agents Chemother 60:5878-84.
8. Kidd SE, Ling LM, Meyer W, Orla Morrissey C, Chen SC, Slavin MA. 2009. Molecular epidemiology of invasive aspergillosis: lessons learned from an outbreak investigation in an Australian hematology unit. Infect Control Hosp Epidemiol 30:1223-6.
9. Camps SM, Rijs AJ, Klaassen CH, Meis JF, O'Gorman CM, Dyer PS, Melchers WJ, Verweij PE. 2012. Molecular epidemiology of *Aspergillus fumigatus* isolates harboring the TR_{34}/L98H azole resistance mechanism. J Clin Microbiol 50:2674-80.
10. Garcia-Rubio R, Escribano P, Gomez A, Guinea J, Mellado E. 2018. Comparison of Two Highly Discriminatory Typing Methods to Analyze *Aspergillus fumigatus* Azole Resistance. Front Microbiol 9:1626.
11. Falahatinejad M, Vaezi A, Fakhim H, Abastabar M, Shokohi T, Zahedi N, Ansari S, Meis JF, Badali H. 2018. Use of cell surface protein typing for genotyping of azole-resistant and -susceptible *Aspergillus fumigatus* isolates in Iran. Mycoses 61:143-147.
12. Sewell TR, Zhang Y, Brackin AP, Shelton JMG, Rhodes J, Fisher MC. 2019. Elevated prevalence of azole resistant *Aspergillus fumigatus* in urban versus rural environments in the United Kingdom. Antimicrob Agents Chemother doi:10.1128/AAC.00548-19.

13. Bader O, Weig M, Reichard U, Lugert R, Kuhns M, Christner M, Held J, Peter S, Schumacher U, Buchheidt D, Tintelnot K, Gross U, MykoLabNet DP. 2013. *cyp51A*-Based mechanisms of *Aspergillus fumigatus* azole drug resistance present in clinical samples from Germany. Antimicrob Agents Chemother 57:3513-7.
14. Bader O, Tunnermann J, Dudakova A, Tangwattanachuleeporn M, Weig M, Gross U. 2015. Environmental isolates of azole-resistant *Aspergillus fumigatus* in Germany. Antimicrob Agents Chemother 59:4356-9.
15. Mushi MF, Buname G, Bader O, Gross U, Mshana SE. 2016. *Aspergillus fumigatus* carrying TR_{34}/L98H resistance allele causing complicated suppurative otitis media in Tanzania: Call for improved diagnosis of fungi in sub-Saharan Africa. BMC Infect Dis 16:464.
16. Tangwattanachuleeporn M, Minarin N, Saichan S, Sermsri P, Mitkornburee R, Gross U, Chindamporn A, Bader O. 2017. Prevalence of azole-resistant *Aspergillus fumigatus* in the environment of Thailand. Med Mycol 55:429-435.
17. Riat A, Plojoux J, Gindro K, Schrenzel J, Sanglard D. 2018. Azole Resistance of Environmental and Clinical *Aspergillus fumigatus* Isolates from Switzerland. Antimicrob Agents Chemother 62.
18. Fan H, Chen Y, Duan L, Zhao J, Qin C, Li H, Sun J, Han L. 2020. Comparison of Two Typing Methods for Characterization of Azole Resistance in *Aspergillus fumigatus* from Potting Soil Samples in a Chinese Hospital. Antimicrob Agents Chemother 64.
19. Chen Y, Dong F, Zhao J, Fan H, Qin C, Li R, Verweij PE, Zheng Y, Han L. 2020. High Azole Resistance in *Aspergillus fumigatus* Isolates from Strawberry Fields, China, 2018. Emerg Infect Dis 26:81-89.
20. Rossler S, Bader O, Stolzel F, Sommer U, Spiess B, Geibel S, Buchheidt D, Gross U, Baretton G, Jacobs E, Ostrosky-Zeichner L. 2017. Progressive Dispersion of Azole Resistance in *Aspergillus fumigatus*: Fatal Invasive Aspergillosis in a Patient with Acute Myeloid Leukemia Infected with an *A. fumigatus* Strain with a *cyp51A* TR_{46}/Y121F/M172I/T289A Allele. Antimicrob Agents Chemother 61:e00270-17.
21. Gonzalez-Jimenez I, Lucio J, Amich J, Cuesta I, Sanchez Arroyo R, Alcazar-Fuoli L, Mellado E. 2020. A Cyp51B Mutation Contributes to Azole Resistance in *Aspergillus fumigatus*. J Fungi (Basel) 6.
